# Clinical, prognostic, and therapeutic significance of heat shock protein 27 in bladder cancer

**DOI:** 10.18632/oncotarget.24091

**Published:** 2018-01-08

**Authors:** Myung-Shin Lee, Jisu Lee, Suhyuk Lee, Seung-Min Yoo, Joo Heon Kim, Won Tae Kim, Wun-Jae Kim, Jinsung Park

**Affiliations:** ^1^ Department of Microbiology and Immunology, Eulji University School of Medicine, Daejeon, South Korea; ^2^ Department of Pathology, Eulji University School of Medicine, Daejeon, South Korea; ^3^ Department of Urology, College of Medicine, Chungbuk National University, Cheongju, South Korea; ^4^ Department of Urology, Eulji University Hospital, Eulji University School of Medicine, Daejeon, South Korea

**Keywords:** bladder cancer, biomarkers, heat shock protein 27, prognosis, shRNA

## Abstract

Heat shock protein 27 (HSP27) is highly expressed in many cancers, and its prognostic and predictive value has been reported. HSP27 knockdown using siRNA or OGX-427 (an anti-sense oligonucleotide sequence targeting HSP27) is reported to have anti-cancer effects and to enhance chemosensitivity of cancer cells to chemotherapeutic agents. However, conflicting results have been reported regarding the clinical significance of HSP27 in bladder cancer (BC). Furthermore, long-term suppression of HSP27 has not been investigated in BC. In this study, we investigated the association between HSP27 expression and BC characteristics in 132 BC patient samples, as well as its prognostic value to determine the potential of HSP27 as a clinical biomarker. Additionally, we applied five different shRNAs targeting HSP27 in three invasive BC cell lines to analyze the long-term knockdown effects of HSP27. Our study revealed a significant association between HSP27 expression and adverse pathological characteristics such as high-stage and -grade BC. However, HSP27 expression was not associated with clinical outcomes such as tumor recurrence, progression, and patient survival. Interestingly, although our shRNAs had obvious knockdown effects on HSP27 in BC cells, we did not find consistent effects on apoptosis of BC cells or chemotherapeutic sensitivity of BC cells to cisplatin. Therefore, although HSP27 may be a predictor of adverse pathological characteristics in BC, its role as a prognostic biomarker and therapeutic target seems to be limited.

## INTRODUCTION

Bladder cancer (BC) is the second most common cancer of the genitourinary tract worldwide [1]. Approximately 15–70% of non-muscle invasive BC (NMIBC) will recur, and a significant proportion of patients with high-risk NMIBC will develop muscle-invasive BC (MIBC) within 5 years [2, 3]. The prognosis of patients with MIBC is poor with current treatments [4–6]. Therefore, identification of novel prognostic biomarkers and development of novel treatments is needed to improve the survival of patients with BC.

Heat shock proteins (HSPs) are molecular chaperones responsible for protein holding and folding of newly synthesized proteins for the maintenance of cellular homeostasis [7]. HSP27 is a small HSP that was initially characterized in a heat shock response, but then was shown to respond to other cellular stress conditions including carcinogenesis [8–10]. HSP27 expression is increased in a variety of malignancies, including colorectal cancer [11], non-small cell lung cancer [12], and hepatocellular carcinoma [13], while its prognostic and predictive value has been reported (nicely reviewed by Ciocca *et al*. [14]). HSP27 overexpression was also reported to be involved in the epithelial-to-mesenchymal transition of lung cancer and prostate cancer cells [15, 16]. However, HSP27 expression in BC has not been investigated extensively, and its clinical and functional roles in BC are controversial. Several studies have reported significant associations between HSP27 expression and BC aggressiveness or patient prognosis [17–19], whereas other studies have shown contradictory results, with no such associations found [20–22].

HSP27 has been also examined as a potential therapeutic target in cancers. For example, OGX-427 (Apatorsen, OncoGenex, Vancouver, BC, Canada), a sequence of second-generation antisense oligonucleotides (ASO) generated using a 2′-O-(2-methoxy) ethyl (2′-MOE) backbone and targeting HSP27 mRNA, was studied in various cancers. OGX-427-mediated HSP27 suppression was reported to inhibit tumor proliferation and sensitize cancer cells to hormone, chemo-, and radiotherapies in hepatocellular carcinoma [13], prostate cancer [23], pancreatic cancer [24], non-small lung cancer [25], and head and neck cancer [26]. Currently, clinical trials are ongoing to confirm the therapeutic effect of OGX-427 in several solid cancers [27]. In line with results in these cancers, a few studies have indicated the therapeutic potential of OGX-427 in BC, based on the induction of apoptosis, enhancement of sensitivity to chemotherapeutic agents, and inhibition of cellular proliferation [19, 28, 29]. However, in those studies suggesting therapeutic effects of OGX-427 in BC [28, 29], OGX-427 alone did not significantly affect BC cell viability or apoptosis, showing only a non-significant trend of tumor suppression. Besides conflicting results regarding the clinical significance of HPS27 in BC [17–22, 28, 29], these findings indicate that although OGX-427 can enhance sensitivity to other chemotherapeutic agents, its therapeutic role is uncertain.

Meanwhile, in our previous study [30] based on an antibody microarray that allowed us to analyze protein expression directly, HSP27 was found to be differentially expressed between NMIBC and MIBC. In addition, given that BC cell phenotype change observed in several studies suggesting therapeutic potential of HSP27 knockdown in BC [19, 28, 29] was dependent on a single OGX-427 siRNA (usually expected to have only transient suppressive effects), we hypothesized that long-term suppression of HSP27 by various shRNA sequences is more effective in inhibiting tumor growth and enhancing chemosensitivity of BC cells. In this study, we sought to investigate the 1) association between HSP27 expression and BC characteristics, 2) prognostic value of HSP27 in BC, and 3) long-term knockdown effects of HSP27 using five different shRNAs in BC cells to determine the therapeutic potential of HSP27.

## RESULTS

### Differential HSP27 expression identified by antibody microarray profiling

**Figure 1:**
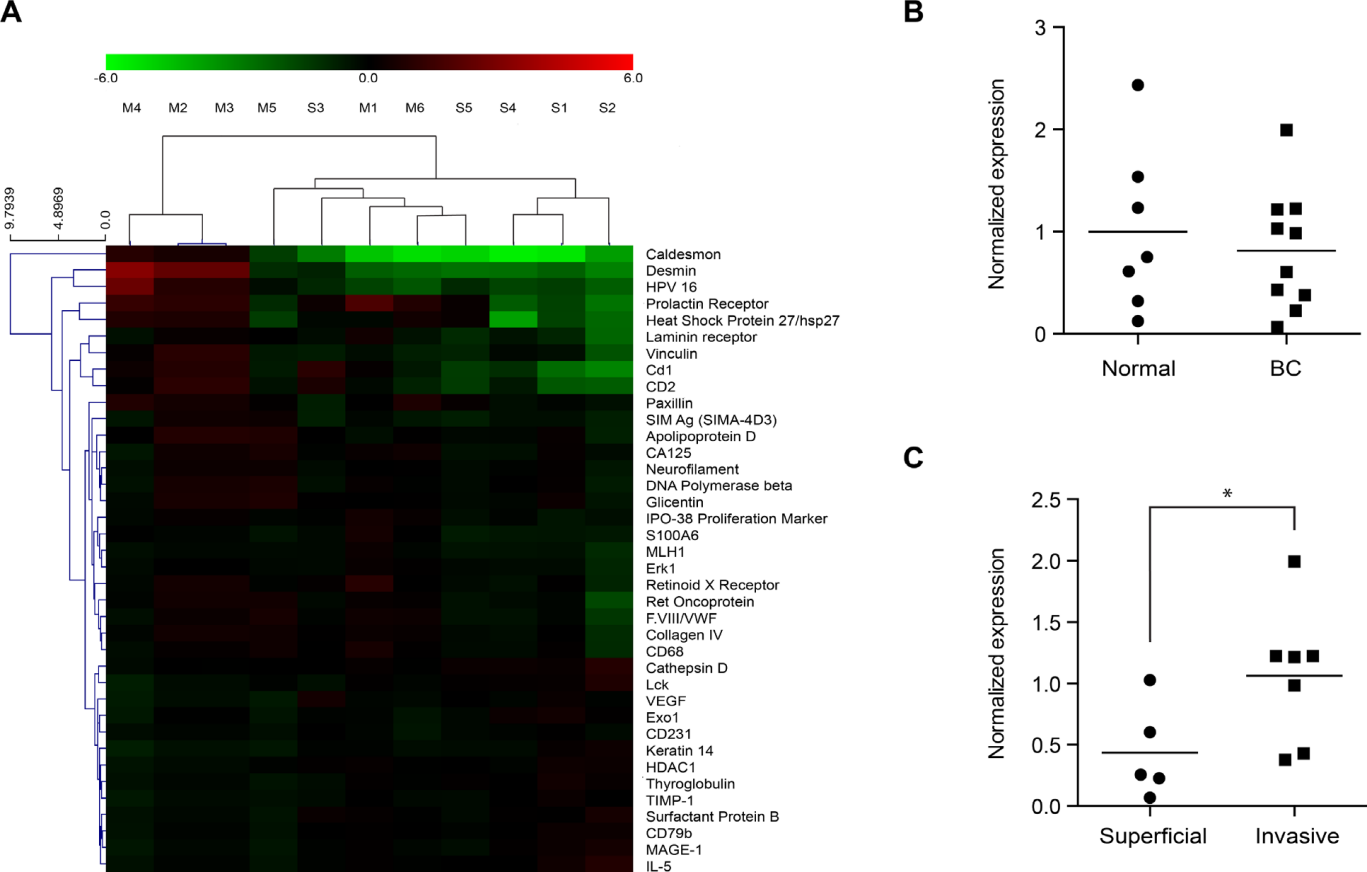
Normalized expression ratio of HSP27 in antibody microarray profiles HSP27 expression in bladder cancer (BC) tissues and normal bladder mucosa were analyzed using data from a previous antibody microarray study [30], which are accessible through GEO Series accession no. GSE69736 (http://www.ncbi.nlm.nih.gov/geo/query/acc.cgi?acc=GSE69736). Briefly, 11 BC tissue samples were obtained from patients with primary non-muscle-invasive BC (NMIBC) (*n* = 5, designated “S”) and muscle-invasive BC (MIBC) (*n* = 6, designated “M”). Seven normal bladder mucosal tissues (designated “normal”) were isolated from the normal bladder mucosa of patients undergoing transurethral resection of bladder tumors (*n* = 3) or were obtained from a tissue biobank (*n* = 4). Protein expression in the 18 tissues samples was analyzed using an antibody microarray kit with 656 antibodies. (**A**) Differential protein expression between primary NMIBC and MIBC tissues. Proteins shown in the right column are those with a > 1.5-fold (or < 0.667) change with *p* values < 0.1. Red indicates higher expression in MIBC tissues as compared to NMIBC; green indicates lower expression in MIBC tissues. Expression of HSP27 in MIBC tissues was significantly higher than that in NMIBC tissues. (**B** and **C**) Statistical analysis for the normalized expression ratios of HSP27 in the antibody microarray profiles (^*^p = 0.042).

Using 11 BC patient samples (five from primary NMIBC and six from MIBC cases) and seven normal bladder mucosal tissues (GEO Series accession no. GSE69736) (Figure 1A) from our previous antibody analysis [30], HSP27 expression was analyzed (Figure 1B and 1C). Although the average normalized expression levels were similar between normal bladder mucosa and BC tissue (Figure 1B, *p* = 0.594), HSP27 expression in MIBC tissues was significantly higher (2.383 fold) than that in NMIBC tissues (Figure 1C, *p* = 0.042). These differential HSP27 protein expressions in BC tissues prompted additional validation studies.

### Expression of HSP27 in human BC cells

**Figure 2:**
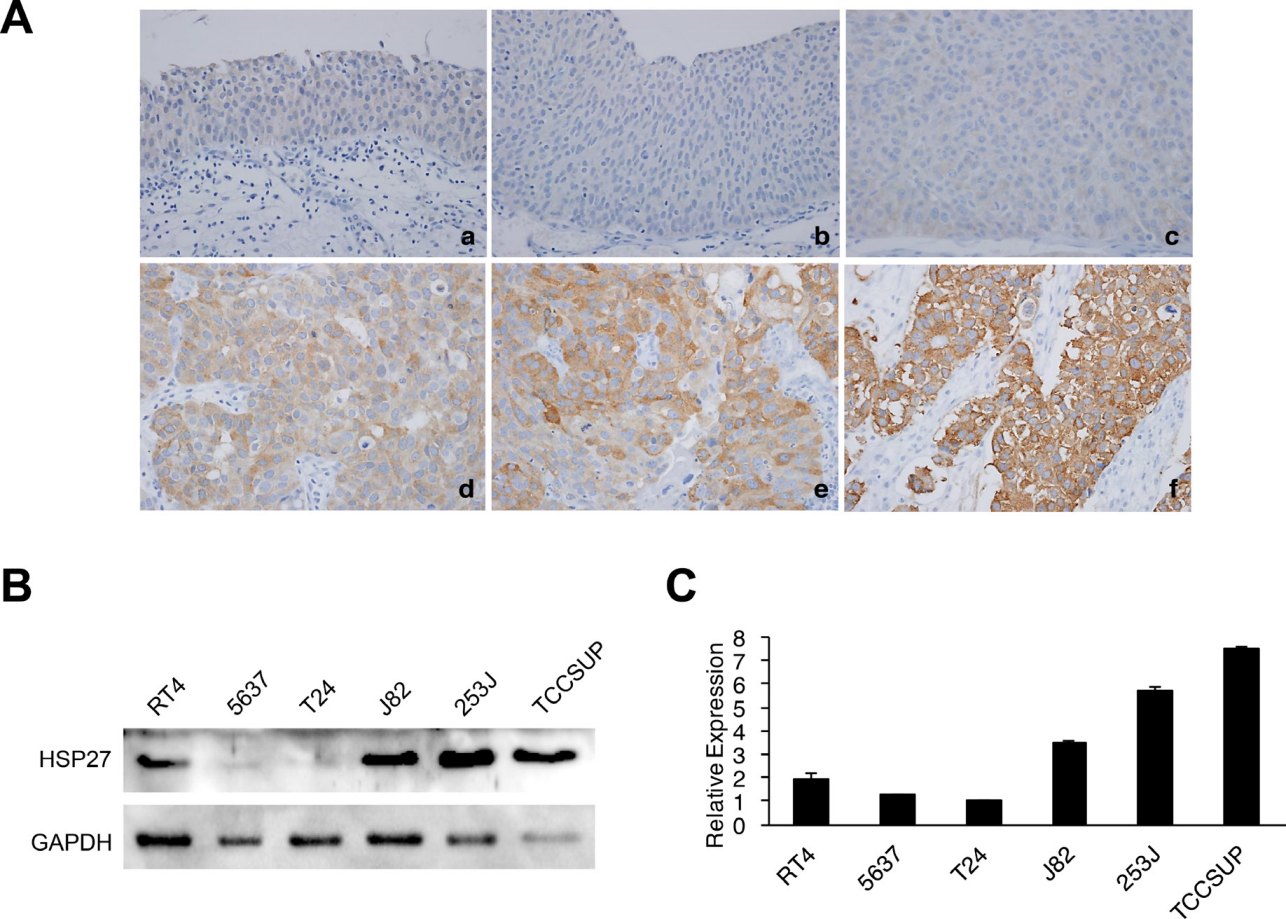
Protein expression of HSP27 in human bladder cancer (BC) tissues and cell lines (**A**) Representative images of HSP27 immunohistochemistry in human BC tissues (magnification, 400×). a. normal urothelium, b. negative expression in low-grade non-muscle-invasive BC (NMIBC), c. negative expression in high-grade NMIBC, d. mild expression in high-grade NMIBC, e. moderate expression in high-grade NMIBC, f. strong expression in high-grade muscle-invasive BC. (**B**–**C**) Total HSP27 protein expression in various BC cell lines was analyzed by western blotting. GAPDH was used as a calibration control. Representative western blots (B). Relative expression was quantified using ImageJ software and is presented in a bar graph (C).

Given the small number of samples in the antibody microarray, the expression of HSP27 in normal urothelial cells and BC was examined in blocks of paraffinized human tissue by immunohistochemistry (Figure 2A). HSP27 was expressed primarily in the cytoplasm of BC cells, but its expression was significantly higher in high-grade MIBC cells compared to that in NMIBC cells, consistent with the results of our antibody microarray profiling study. However, HSP27 expression was absent or very weak in normal urothelial cells (Figure 2A). Inconsistent results of HSP27 expression by antibody microarray profiling and immunohistochemistry in normal bladder mucosa are thought to be because of the inclusion of a stromal component in the antibody microarray, as discussed in our prior report [30]. The expression of HSP27 in BC cells was also analyzed by western blot (Figure 2B and 2C). Although expression varied among cells, BC cells with higher invasive potential showed higher expression of HSP27, which is consistent with results of the antibody microarray. Expression of mRNA in BC cells was also consistent with protein expression (Supplementary Figure 1). Three BC cell lines (J82, 253J, and TCCSUP) that show high expression of HSP27 were chosen for the experiment of shRNA knockdown study.

### Association between immunohistochemical HSP27 expression and clinicopathological characteristics

To validate the relevance of HSP27 as a clinical biomarker in BC, HSP27 expressions were analyzed with 132 human NMIBC tissues by immunohistochemistry. Baseline characteristics of a validation cohort comprising 132 patients are shown in Supplementary Table 1. The median age of the patients was 68 (range 28–85) years. The immunohistochemical scores, based on staining area and intensity, were as follows: HSP27 expression was negative in 49 patients (37.1%), mild in 44 (33.3%), moderate in 36 (27.3%), and strong in 3 (2.3%), respectively. Based on these data, HSP27 expression was dichotomized as negative versus ≥ mild (designated as “positive”), because such grouping showed the most significant survival difference in the Kaplan–Meier analysis. Positive expression of HSP27 was associated with adverse clinicopathological characteristics such as larger tumor size, lymphovascular invasion, intravesical therapy, higher T stage and grade (Table 1).

**Table 1:** Association between heat shock protein 27 (HSP27) expression and clinicopathological characteristics

Variables	HSP27 expression^*^		
	Negative	Positive	*p*
Total no. (%)	49 (37.1)	83 (62.9)	−
Gender (no. [%])			0.493
Male	38 (77.6)	69 (83.1)	
Female	11 (22.4)	14 (16.9)	
Tumor size (no. [%])			**0.048**
<3cm	40 (81.6)	54 (65.1)	
≥3cm	9 (18.4)	29 (34.9)	
Multifocality (no. [%])			0.058
Single	42 (85.7)	58 (69.9)	
Multiple	7 (14.3)	25 (30.1)	
Concomitant carcinoma-*in-situ* (no. [%])			0.054
No	45 (91.8)	65 (78.3)	
Yes	4 (8.2)	18 (21.7)	
Morphology (no. [%])			0.414
Papillary	45 (91.8)	72 (86.7)	
Sessile	4 (8.2)	11 (13.3)	
Lymphovascular invasion (no. [%])			**0.034**
No	45 (91.8)	63 (75.9)	
Yes	4 (8.2)	20 (24.1)	
Intravesical therapy (no. [%])			**<0.001**
No	37 (75.5)	34 (41.0)	
Yes	12 (24.5)	49 (59.0)	
T stage (no. [%])			**<0.001**
Ta	39 (79.6)	18 (21.7)	
T1	10 (20.4)	65 (78.3)	
Grade (no. [%])			**<0.001**
Low	40 (81.6)	37 (44.6)	
High	9 (18.4)	46 (55.4)	

^*^The immunohistochemical score was based on both staining area and intensity, and HSP27 expression was negative in 49 patients (37.1%), mild in 44 (33.3%), moderate in 36 (27.3%), and strong in 3 (2.3%), respectively. Its expression was dichotomized (negative vs. positive) because such grouping showed the most significant survival difference on the Kaplan-Meier analysis.

### Prognostic significance of HSP27 expression

The median follow-up time of patients in the validation cohort was 48.4 (mean 52.2, range 6–148.6) months. During surveillance, 46 (34.8%) and 19 (14.4%) of the patients experienced recurrence and progression at a median time of 14.1 mo. and 33.9 mo., respectively. In the Kaplan-Meier survival analysis, positive HSP27 expression was significantly associated with lower recurrence-free survival relative to that with negative expression (*p* = 0.003), but it was not associated with progression-free survival (*p* = 0.064). Similarly, univariate Cox regression analyses showed that HSP27 expression was a significant predictor for tumor recurrence (*p* = 0.004, 95% confidence interval 1.396–6.091) but not for progression (*p* = 0.072, 95% confidence interval 0.902–8.387) (Table 2). However, a multivariate analysis adjusting for HSP27 expression and multiple clinicopathological factors showed that HSP27 expression was not predictive of tumor recurrence (*p* = 0.094, 95% confidence interval 0.881–5.024) or progression (*p* = 0.144, 95% confidence interval 0.720–9.516) (Table 2). In different multivariate models, including those with various sets of clinicopathological parameters, HSP27 was not associated with tumor recurrence or progression. For example, in a multivariate model without including intravesical therapy (Table 2), HSP27 was not predictive of tumor recurrence or progression in NMIBC.

**Table 2:** Univariate and multivariate Cox regression analyses of multiple variables for recurrence and progression-free survival

Variables	Recurrence-free survival			Progression-free survival		
	HR	95% CI	*p*	HR	95% CI	*p*
**Univariate analysis**						
Age (as continuous variable)	1.023	0.998 – 1.049	0.069	1.045	1.001 – 1.091	**0.043**
Gender (male vs female)	0.966	0.465 – 2.005	0.925	1.687	0.639 – 4.449	0.291
Tumor size (<3 cm vs ≥3 cm)	2.080	1.155 – 3.744	**0.015**	1.859	0.741 – 4.664	0.186
Multifocality (single vs multiple)	2.106	1.146 – 3.870	**0.016**	1.272	0.458 – 3.536	0.645
Concomitant carcinoma-in-situ (no vs yes)	1.242	0.598 – 2.584	0.561	4.842	1.903 – 12.320	**0.001**
Morphology (papillary vs non-papillary)	0.883	0.346 – 2.255	0.795	2.592	0.824 – 8.155	0.103
Lymphovascular invasion (no vs yes)	1.447	0.717 – 2.919	0.302	1.837	0.658 – 5.125	0.246
Intravesical therapy (no vs yes)	0.684	0.378 – 1.238	0.210	0.375	0.135 – 1.043	0.060
T stage (Ta vs T1)	3.154	1.557 – 6.389	**0.001**	2.431	0.868 – 6.810	0.091
Grade (low vs high)	2.276	1.254 – 4.128	**0.007**	2.505	0.981 – 6.395	0.055
HSP27 expression^*^ (negative vs positive)	2.916	1.396 – 6.091	**0.004**	2.750	0.902 – 8.387	0.075
**Multivariate analysis (Model including intravesical therapy)**						
Age (as continuous variable)	1.015	0.987 – 1.043	0.302	1.023	0.975 – 1.073	0.359
Tumor size (<3 cm vs ≥3 cm)	1.496	0.778 – 2.876	0.227	1.546	0.558 – 4.279	0.402
Multifocality (single vs multiple)	4.053	1.793 – 9.162	**0.001**	2.420	0.618 – 9.474	0.204
Concomitant carcinoma-*in-situ* (no vs yes)	0.445	0.197 – 1.005	0.051	3.201	0.931 – 11.010	0.065
Intravesical therapy (no vs yes)	0.188	0.077 – 0.456	**< 0.001**	0.160	0.036 – 0.706	**0.016**
T stage (Ta vs T1)	1.991	0.819 – 4.839	0.128	1.074	0.293 – 3.934	0.914
Grade (low vs high)	2.811	1.320 – 5.990	**0.007**	1.428	0.338 – 6.035	0.628
HSP27 expression^*^ (negative vs positive)	2.103	0.881 – 5.024	0.094	2.617	0.720 – 9.516	0.144
**Multivariate analysis (Model without including intravesical therapy)**						
Age (as continuous variable)	1.025	0.997 – 1.055	0.079	1.038	0.991 – 1.087	0.112
Tumor size (<3 cm vs ≥3 cm)	1.683	0.898 – 3.153	0.104	1.651	0.631 – 4.318	0.307
Multifocality (single vs multiple)	1.789	0.909 – 3.518	0.092	0.854	0.291 – 2.505	0.773
Concomitant carcinoma-*in-situ* (no vs yes)	0.595	0.266 – 1.330	0.205	4.448	1.285 – 15.401	**0.018**
T stage (Ta vs T1)	2.036	0.913 – 4.839	0.082	1.211	0.382 – 3.835	0.745
Grade (low vs high)	1.693	1.320 – 5.990	0.135	0.787	0.214 – 2.892	0.718
HSP27 expression^*^ (negative vs positive)	1.526	0.881 – 5.024	0.310	1.934	0.562 – 6.656	0.296

^*^The immunohistochemical score was based on both staining area and intensity, and HSP27 expression was negative in 49 patients (37.1%), mild in 44 (33.3%), moderate in 36 (27.3%), and strong in 3 (2.3%), respectively. Its expression was dichotomized (negative vs. positive) because such grouping showed the most significant survival difference on the Kaplan-Meier analysis.

Besides NMIBC, we also analyzed the prognostic value of HSP27 expression in MIBC. In our cDNA microarray database including MIBC [31], we found that HSPB1 (gene for HSP27) expression was not associated with cancer-specific survival (*p* = 0.522) and overall survival (*p* = 0.488) of MIBC patients (Supplementary Figure 2A and 2B). To externally validate our findings, we analyzed the prognostic value of HSP27 in an independent TCGA database [32]. We confirmed that HSPB1 expression had no prognostic value in predicting disease-free survival (*p* = 0.826) and overall survival (*p* = 0.690) in MIBC patients (Supplementary Figure 3A and 3B).

### Suppression of HSP27 expression by shRNA

**Figure 3:**
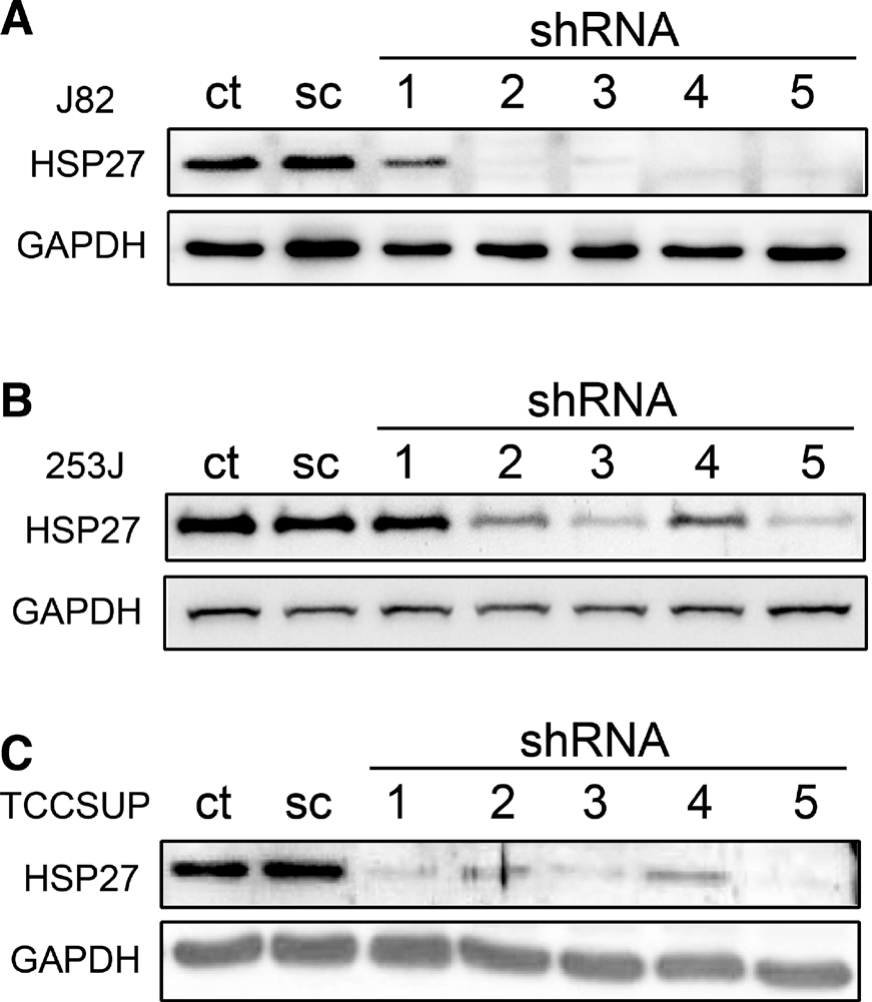
shRNA-mediated knockdown of HSP27 in BC cells BC cells were transfected with HSP27 shRNAs by lentivirus infection. shRNA-transfected J82 (**A**), 253J (**B**), and TCCSUP cells (**C**) were isolated by 3 weeks of puromycin selection, and HSP27 expression in each cell line was analyzed by western blot. GAPDH was used as a housekeeping protein control. ct: non-transfected control; sc: scramble shRNA; shRNA: shRNA against HSP27.

Five plasmids containing different shRNA sequences for human HSP27 were inoculated into three BC cell lines, J82, 253J, and TCCSUP, using a lentivirus-mediated infection system. After more than 3 weeks of selection of the transfected cells with puromycin, HSP27 expression was analyzed (Figure 3). The cell lines showed different knockdown efficiencies. In J82 and 253J, shRNA1 showed little suppression on HSP27. However, obvious suppression of HSP27 by the shRNAs was observed in the other cell lines. Interestingly, the same shRNA had different effects on HSP27 expression in the various BC cell lines.

### Viability of BC cells with HSP27 knocked down

**Figure 4:**
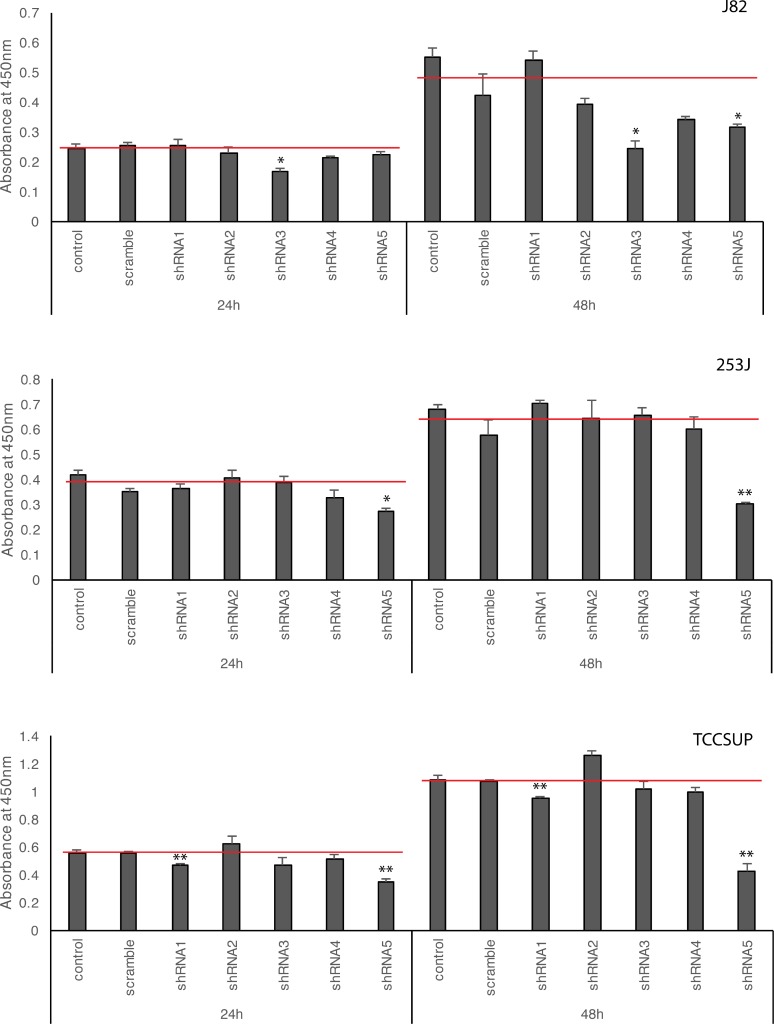
Effects of HSP27 knockdown on BC cell proliferation The same numbers of shRNA-transfected BC cells were seeded onto a 96-well culture plate. The proliferation of BC cells transfected with shRNA as measured by WST-1 assay at 24 and 48 h. ct: non-transfected control, scramble: scramble shRNA, shRNA1-5: shRNA for HSP27. ^*^p < 0.05, ^**^p < 0.01, Student's *t*-test. Red lines in the graph indicate mean value of BC proliferation after transfection of control and scramble shRNA plasmid.

**Figure 5:**
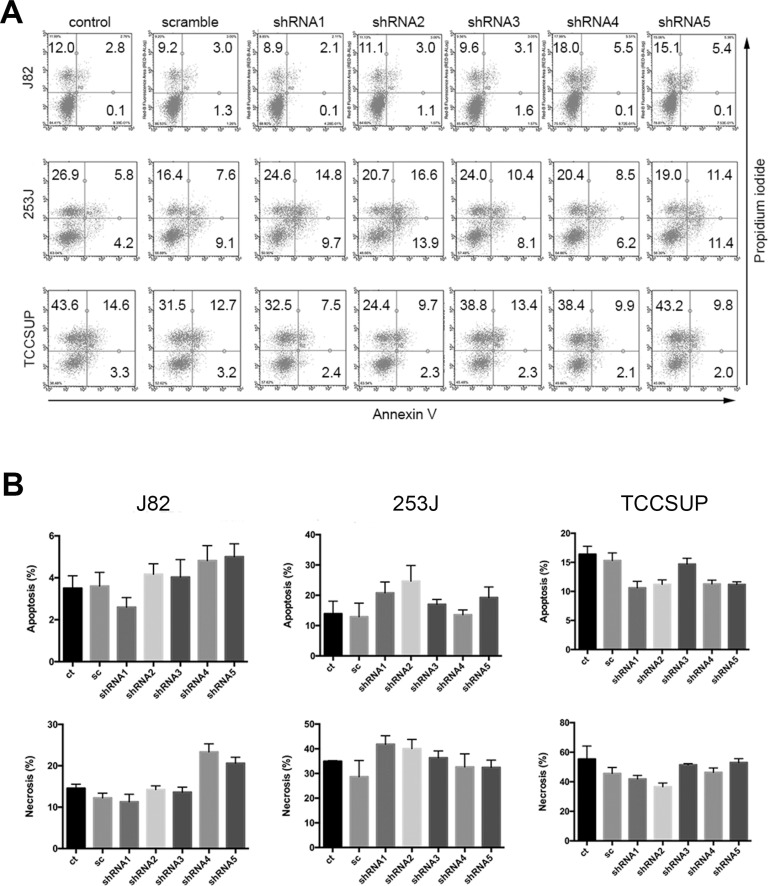
Effects of HSP27 knockdown on BC cell apoptosis and death shRNA-transfected BC cells were cultured in serum-free culture media for 48 h. Apoptosis and necrosis of these cells were analyzed by staining with Annexin V and propidium iodide (PI). (**A**) Representative staining results from three independent experiments. The percentages of Annexin V- and PI-positive cells are indicated. (**B**) The average percentages of cells showing apoptosis and necrosis from (A) are indicated. ct: non-transfected control, sc: scramble shRNA, shRNA1-5: shRNA for HSP27.

To analyze the effect of HSP27 knockdown on BC cell proliferation, a WST-1 cell proliferation assay was performed after five shRNA transfections in the three BC cell lines (Figure 4). In J82, shRNA3 and shRNA5 significantly inhibited cell growth. shRNA1, which had a weak suppressive effect on HSP27 expression, did not show any effect on proliferation. shRNA2 and shRNA4 slightly inhibited BC cell growth, but this effect was not significant. In 253J, only shRNA5 showed significant inhibition of proliferation. In TCCSUP, only shRNA1 and shRNA5 inhibited proliferation significantly; however, other shRNAs also showed obvious suppression of HSP27 expression (Figure 3C). Taken together, only shRNA5 for HSP27 showed consistent inhibition of cell proliferation in the three BC cell lines; therefore (integrating Figure 3 and Figure 4), it was difficult to conclude that silencing of HSP27 has an anti-proliferative effect on BC cells. Because a previous study indicated that OGX-427 treatment induced apoptosis in UMUC-3 BC cells [19], we also analyzed apoptosis and cell death. However, in our normal culture conditions, all shRNA-transfected BC cells were healthy and only a few dead cells were observed. Therefore, we analyzed apoptosis in BC cells cultured with serum-free media for 48 h (Figure 5). Although a few shRNA-knockdown BC cells showed more apoptosis or necrosis than scramble shRNA-treated BC cells, we found no significant and consistent effect on the viability of HSP27-knockdown BC cells.

### Association of HSP27 expression and chemosensitivity of BC cells

**Figure 6:**
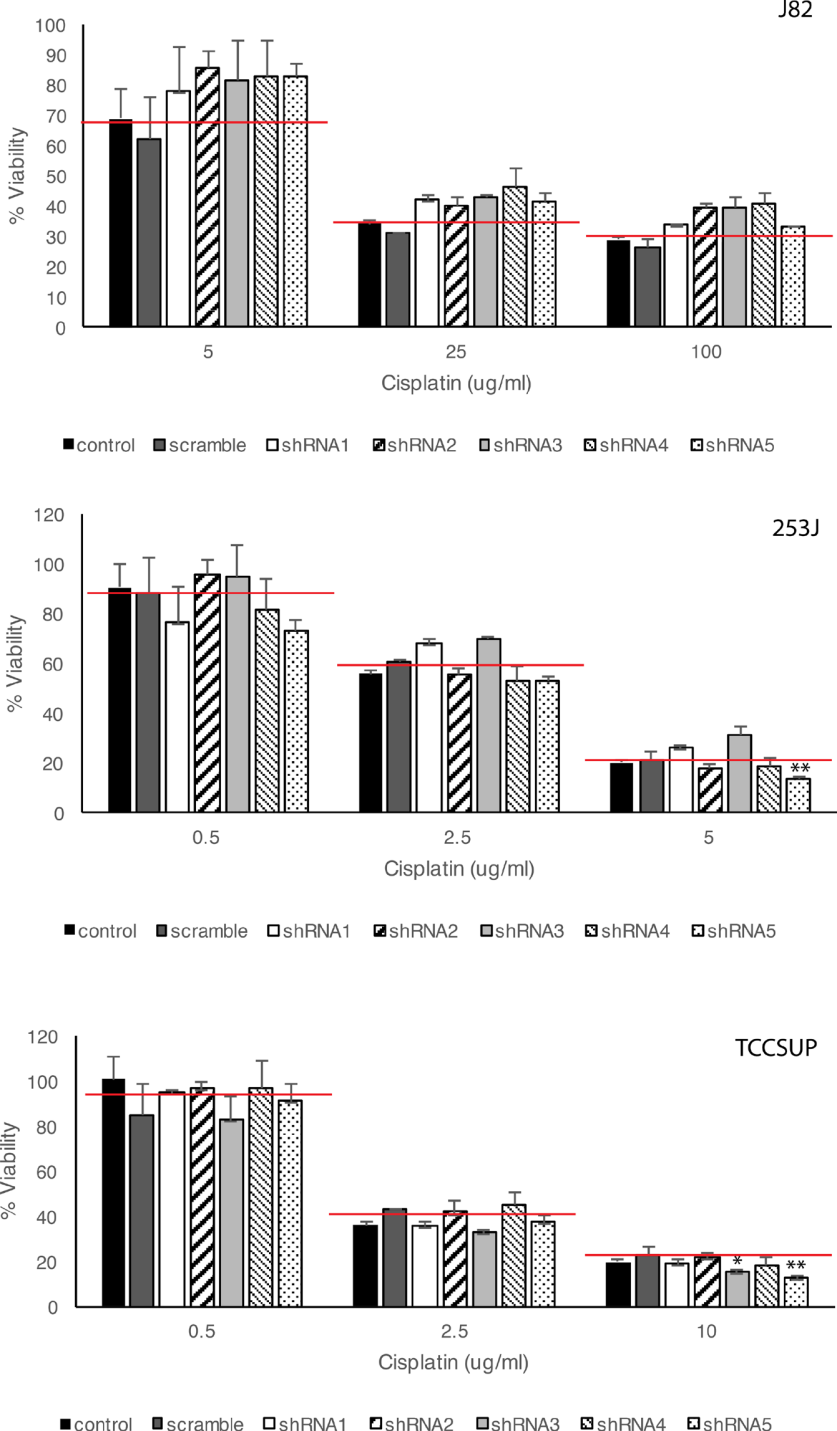
Effects of HSP27 knockdown on cisplatin-induced BC cell death ShRNA-transfected BC cells were incubated with various concentrations of cisplatin for 48 h. Cell viability was analyzed by WST-1 assay. ct: non-transfected control, scramble: scramble shRNA, shRNA1-5: shRNA for HSP27. ^*^p < 0.05, ^**^p < 0.01, Student's *t*-test. Red lines in the graph indicate mean value of BC cell viability after transfection of control and scramble shRNA plasmid.

To analyze the chemotherapeutic sensitivity of BC cells to cisplatin according to HSP27 expression, apoptosis and death of cells cultured in various cisplatin concentrations were examined after HSP27 knockdown using five shRNAs (Figure 6). To evaluate the dose-dependent effects of cisplatin, cell viability was analyzed with three different concentrations of the chemotherapeutic. Higher dose of cisplatin induced more cell death. However, HSP27-knockdown cells did not show significant differences in their chemotherapeutic sensitivity at the same concentration of cisplatin from that of HSP27-expressing cells. shRNA3 or shRNA5 resulted in enhanced chemosensitivity of 253J and TCCSUP cells at the highest cisplatin concentration. However, because HSP27 suppression did not increase sensitivity to cisplatin in J82 and other shRNAs did not show significant effects on the chemosensitivity of 253J or TCCSUP cells, the association between HSP27 and chemosensitivity was not consistent among all BC cell lines.

## DISCUSSION

Existing evidence indicates the diagnostic, prognostic, and therapeutic significance of HSP27 in various cancers [11–14, 33–36]. However, HSP27 has not been thoroughly examined in BC, and its clinical significance remains controversial. To our knowledge, this is the first study to investigate clinicopathological and prognostic significance of HSP27 expression in NMIBC, as well as the therapeutic potential of long-term knockdown of HSP27 in MIBC.

We found a significant association between HSP27 expression and adverse clinicopathological characteristics such as higher stage and grade in both BC cells (Figure 1 and Figure 2) and specimens from a large NMIBC cohort (Table 1); however, we did not confirm its prognostic value in NMIBC (Table 2). Results of several studies regarding the prognostic potential of HSP27 have conflicted. For example, in an N-butyl-N-(4-hydroxybutyl) nitrosamine-induced BC animal model, HSP27 was overexpressed in hyperplastic tissue relative to that in control tissues [17]. In 75 schistosomiasis-associated BC cases, 45 (60%) patients showed HSP27 expression, and a significant correlation was found between expression of HSP27 and tumor grade, stage, and recurrence [18]. Similarly, the Vancouver group reported that HSP27 overexpression increased BC cell growth and that downregulation of HSP27 induced apoptosis of BC cells [19]. However, results of other studies do not support these results. In a US study of 24 MIBC cystectomy specimens, HSP27 did not correlate with tumor characteristics, recurrence, or survival [20]. A French study based on immunohistochemistry of 42 (37 NMIBC and 5 MIBC) tissue sections reported that HSP27 was not associated with tumor grade and, notably, that low expression of HSP27 correlated with a higher tumor stage [21]. Similarly, in a study of 744 (530 NMIBC and 214 MIBC) tissue samples, low expression of HSP27 correlated with poor prognosis [22]. The exact reasons for these conflicting results remain unclear. However, it is noteworthy that most prior studies on the clinicopathological and prognostic significance of HSP27 expression in BC were based on a small number of patients [18, 20, 21] and included either an animal model [17] or BC cell lines [19] only, without examining patient-derived samples.

Importantly, although HSP27 expression was significantly associated with clinicopathological characteristics of BC, we did not find any prognostic value of HSP27 expression. To date, only a few studies have investigated the prognostic value of HSP27 in BC, and these results are also conflicting [18, 20, 22]. Of note, an Egyptian study [18] included patients with squamous cell carcinoma (not urothelial carcinoma), and a US study included only 24 muscle-invasive BC specimens, thus limiting the statistical analyses. Although a Taiwanese study [22] reported an association between lower HSP27 expression and poorer prognosis in BC, the results were based on a tissue microarray (not whole specimens) with different immunohistochemistry cut-off values from ours. We found that HSP27 expression was not significant for tumor recurrence or progression in NMIBC on multivariate analyses adjusting multiple clinicopathological parameters (Table 2). Similar to NMIBC, we did not find prognostic value of HSP27 in MIBC (Supplementary Figure 2). Also, external validation in an independent TCGA database (Supplementary Figure 3) supports our findings. Further studies are needed to elucidate the exact reasons of conflicting results among studies.

The therapeutic potential of modulating HSP27 expression in MIBC was another main focus in our study. To our knowledge, we are the first to demonstrate the inhibition of BC cell proliferation by shRNA-mediated long-term suppression of HSP27. In contrast to previous study using siRNA or ASO for HSP27 [19, 28, 29], we did not find a conclusive effect of HSP27 suppression on apoptosis or chemosensitivity of BCs, which suggests that the use of HSP27 as a therapeutic target for BCs should be approached carefully.

Because of diverse cellular functions and cancer associations, HSP27 has been reported as a therapeutic target for cancer treatments. OGX-427 is the most well-known ASO to inhibit HSP27 expression, and it has been suggested to have therapeutic effects in BC [19, 28, 29]. However, the functional significance of HSP27 suppression was assessed only with OGX-427, and long-term suppression of HSP27 was not investigated. In the present study, we suppressed HSP27 expression using five different shRNA sequences to analyze the functional role of HSP27 in BC cells. Although cellular proliferation was inhibited by some shRNAs, apoptosis and chemosensitivity were not affected by HSP27 knockdown in our experimental conditions. Because three BC cell lines showed similar effects by HSP27 suppression, our results are not specific for a single cell line. Although OGX-427 was reported to enhance sensitivity to chemotherapeutic agents in BC cells [19, 28, 29], shRNA suppression of HSP27 had little effect on sensitivity to cisplatin. In our results, transfection with one or two shRNAs enhanced chemosensitivity of some BC cells, but this effect was not consistent with the extent of HSP27 suppression, suggesting this finding might not be specific to HSP27 knockdown. Inconsistency between our results and those of previous studies might be caused by off-target effect: i.e. an ASO or RNA interference will inevitably have partial complementarity to non-target transcripts, and this may cause suppression of unintended off-target genes [37]. The sequence of the shRNAs used in this study is completely different from that of the OGX-427. While the OGX-427 sequence corresponds to the human HSP27 translation initiation site [25, 29], the shRNAs used in our study targeted the middle to end of HSP27. It is difficult to determine all of the possible off-target effects of each siRNA or shRNA. However, in our study, only one shRNA (shRNA5) among five shRNAs against HSP27 showed anti-proliferative effects in all three BC cells, which indicate that the effect of shRNA5 is more likely to be due to the off-target effect than to the knockdown of HSP27. Notably, we found that prior several studies suggesting therapeutic effects of OGX-427 in BC [28, 29], OGX-427 alone did not significantly affect BC cell viability or apoptosis, showing only a non-significant trend of tumor suppression. To elucidate the precise effects of HSP27 in BC, further studies, including *in vivo* or micro-environmental studies, would be needed.

In summary, we found a significant association between HSP27 expression and adverse BC pathological characteristics such as higher stage and grade in both BC cells and specimens from a large cohort of patients with BC. However, our results, based on survival analyses in BC patients and long-term knockdown of HSP27 in MIBC cells *in vitro,* indicate that HSP27 expression has limited clinical value as a prognostic biomarker or therapeutic target. Further studies focusing on the functional mechanisms associated with HSP27 will be needed to explain the conflicting results from various studies.

## MATERIALS AND METHODS

In the present study, we focused on urothelial carcinomas, hereafter called BC, and excluded other BC histologic variants (squamous, micropapillary, sarcomatoid, small cell, and adenocarcinoma).

The study protocol was approved by the Institutional Review Board of the Eulji University Hospital (approval no. 2011–0081), and informed consent was obtained in all cases. Tumors were staged and graded according to the 7th American Joint Committee on Cancer criteria and 2004 World Health Organization grading system. All surgeries were performed with a curative intent using a previously described standard technique [38].

### Cell culture and reagents

BC cell lines (RT4, 5637, T24, J82, 253J, and TCCSUP) were obtained from the Korean Cell Line Bank (Seoul, South Korea) or American Type Culture Collection (ATCC, Rockville, MD). All cells were cultured in Eagle's minimum essential medium (Corning Life Science, Corning, NY) supplemented with 10% fetal bovine serum (FBS, Gibco BRL, Grand Island, NY) and 1% penicillin-streptomycin (Sigma, St. Louis, MO). The cells were maintained in a humidified atmosphere of 5% CO_2_ at 37°C.

### Lentivirus infections

**Figure 7:**
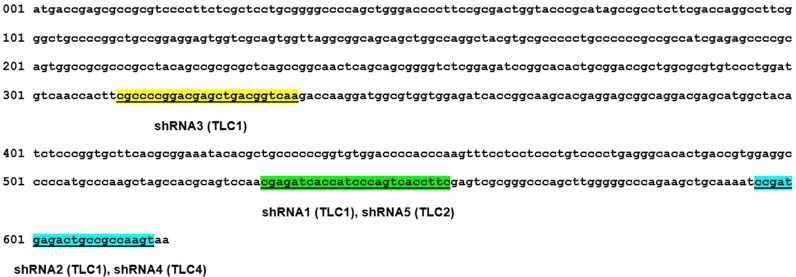
Schematic representation of shRNA-targeted sequences in HSP27 HSP27 shRNA-targeted sequences are underlined and highlighted in the human HSP27 CDR region for each shRNA. TLC1: sigma TLC1 vector based shRNA. TLC2: Sigma TLC2 vector based shRNA.

Plasmids containing shRNAs for human HSP27 (TRCN0000008752, TRCN0000008753, TRCN0000011466, TRCN0000342857, and TRCN0000342790, Sigma) or a scramble shRNA (#1864, Addgene, Cambridge, MA) were co-transfected with pVSV-G and a packaging plasmid (SBI, Palo Alto, CA) into HEK293T cells using Lipofectamine 3000 transfection reagent (Thermo Scientific, Waltham, MA). The HSP27-targeted sequence of each shRNA is shown schematically in Figure 7. TRCN0000008752, TRCN0000008753, TRCN0000011466, TRCN0000342857, and TRCN0000342790 were designated shRNA1, shRNA2, shRNA3, shRNA4, and shRNA5, respectively. BC cell lines were inoculated with viral supernatants from HEK293T cells and polybrene (5 μg/ml) for 48 h. After 10 days of selection with puromycin (1.5 μg/ml), the efficiency of HSP27 knockdown was evaluated by western blotting.

### Western blot

Total proteins were isolated using a 1× sodium dodecyl sulfate (SDS) buffer containing 62.5 mM Tris-HCL at pH 6.8, 2% w/v SDS, 10% v/v glycerol, 50 mM dithiothreitol, and 0.01% w/v bromophenol blue. The cell suspension was boiled for 10 min and then centrifuged at 13,000 rpm for 8 min. The proteins were resolved by electrophoresis in a 12% SDS polyacrylamide gel and transferred to a nitrocellulose membrane (GE Healthcare, Pittsburgh, PA). The membranes were blocked with 5% skim milk in Tris-buffered saline with 0.1% Tween 20 (TBST). Polyclonal rabbit anti-HSP27 (Santa Cruz Biotechnology, Dallas, TX) and polyclonal rabbit anti-GAPDH (Cusabio, College Park, MD) were used as primary antibodies. HRP-conjugated anti-rabbit antibodies (Santa Cruz Biotechnology) were used as secondary antibodies. The results were visualized using an ECL detection reagent (Bio-Rad, Hercules, CA) and a ChemiDoc Touch Imaging System (Bio-Rad).

### Cell proliferation assay

Cell viability was measured using WST-1 cell proliferation reagent (Roche Applied Sciences, Indianapolis, IN). About 1 × 10^4^ cells/well were introduced into 96-well plates and incubated overnight. Following incubation in culture medium or treatment with the indicated concentration of cisplatin (Sigma) for 48 h, the culture medium was replaced by medium containing WST-1 (1:10 dilution) and incubated for 30 min at 37°C in an incubator. The optical density was measured at 450 nm and 650 nm using a microplate reader (Molecular Devices, Sunnyvale, CA). The data are representative of three independent experiments performed in triplicate.

### Apoptosis assay

To detect apoptosis and cell death, cells were detached from the plate by trypsinization and stained using an Annexin V apoptosis detection reagent labeled with allophycocyanin (eBioscience, San Diego, CA) according to the manufacturer's recommendations. A Guava easyCyte Flow Cytometer and InCyte 3.1 software (Merck Millipore, Bedford, MA) were used for the analysis.

### Pathology evaluation and immunohistochemistry

All pathology slides were thoroughly re-evaluated by a single uropathologist (JHK). Immunohistochemistry was performed on tissue specimens, including those from BC and normal urothelium. In addition, to validate the prognostic value of HSP27 in primary NMIBC, an immunohistochemical analysis was performed using specimens from an independent primary NMIBC cohort comprising 132 patients at the Eulji University Hospital. The patients were monitored according to our follow-up protocol [38, 39]. Patients with short-term follow-up periods (less than 6 months) were excluded.

Immunohistochemical staining and evaluation was conducted as described in our previous reports [30, 39, 40], using polyclonal rabbit anti-HSP27 antibody (Santa Cruz Biotechnology, Dallas, TX). The optimal primary antibody dilution was predetermined using appropriate positive (breast cancer tissues) and negative (omission of primary antibody) controls. Immunoreactivity was evaluated by light microscopy twice, at 4-week intervals, by a single uropathologist (JHK) who was blinded to clinical outcomes. Repeat readings of the same samples showed high concordance (κ = 0.842, *p* < 0.001). Immunoreactivity was evaluated semi-quantitatively by integrating staining intensity and the percentage of positively stained cells, as described in our prior reports [30, 40], and an immunohistochemical score was calculated by multiplying the intensity scores and staining area. Scores indicated negative (0–1), mild (2–3), moderate (4–8), or strong (9–12) expression.

### Statistical analysis

All results are shown as means ± standard deviations. Chi-square tests were used to evaluate the association between categorical variables. The one-tailed Student's *t* test was used to assess the significance of differences between groups. To validate the prognostic value of HSP27, recurrence-free and progression-free survival rates were analyzed. Tumor recurrence was defined as the presence of pathological evidence of similar- or lower-stage disease by bladder biopsy or transurethral resection of bladder tumors, and progression was defined as a pathological shift to more advanced stage disease. The Kaplan-Meier method was used to calculate recurrence-free and progression-free survival rates, and differences were evaluated using the log-rank test.

For analysis, measures of HSP27 expression based on immunohistochemical scores were dichotomized appropriately because these groupings showed the most significant differences in the survival analysis. The prognostic significance of HSP27 expression was assessed using univariate and multivariate Cox regression analyses models. *P*-values < 0.05 were considered statistically significant. All statistical analyses were performed using Stata/SE version 12.1 (Stata Corporation, College Station, TX).

## SUPPLEMENTARY MATERIALS FIGURES AND TABLES


