# Supplementary material for: Clinical, prognostic, and therapeutic significance of heat shock protein 27 in bladder cancer
Source: Oncotarget. 2018 Jan 8;9(8):7961–74. doi: 10.18632/oncotarget.24091 (PMC5814273; doi:10.18632/oncotarget.24091)
Supplement: Supplementary file 1 [file oncotarget-09-7961-s001.pdf]

Clinical, prognostic, and therapeutic significance of heat shock protein 27 in bladder cancer

SUPPLEMENTARY MATERIALS

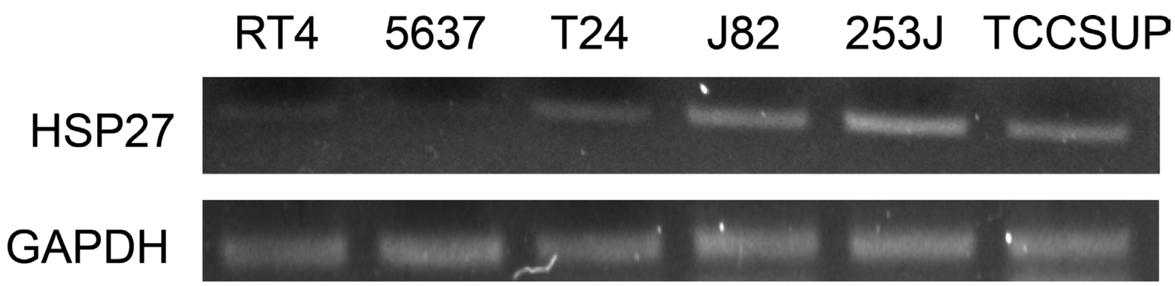

Supplementary Figure 1: mRNA expression of HSP 27 was analyzed by RT-PCR in several human bladder cancer cell lines. Total RNA was prepared using a TRI-reagent as recommended by the manufacturer (Sigma). Total RNA was reverse transcribed using the SuperScript III First-Strand Synthesis System (Thermo Scientific) and PCR was performed using BioFACT 2X Taq PCR Master Mix (BioFACT, Daejeon, South Korea). The cycling conditions were as follows: 95 °C for 2 min; 30 cycles of 95 °C for 20 s, 55 °C for 20 s and 72 °C for 30 s followed by a final elongation step at 72 °C for 10 min. GAPDH mRNA levels were used for normalization. The primers were: HSP27_S 5′-TCCAACGAGATCACCATC-3′, HSP27_AS 5′-AGTCTCATCGGATTTTGC-3′, GAPDH_S 5′-GGTATCGTGGAAGGACTC-3′, GAPDH_AS 5′-GTAGAGGCAGGGATGATG-3′.

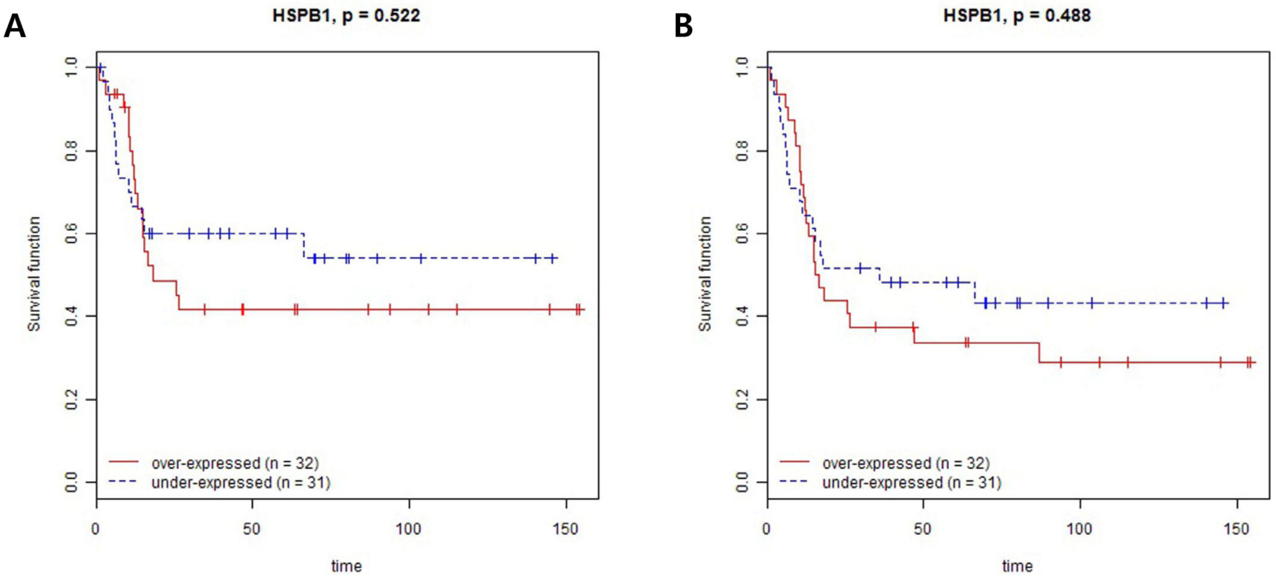

Supplementary Figure 2: Kaplan–Meier survival curves for cancer-specific survival (**A**) and overall survival (**B**) according to HSPB1 gene expression in muscle-invasive bladder cancer [31].

A

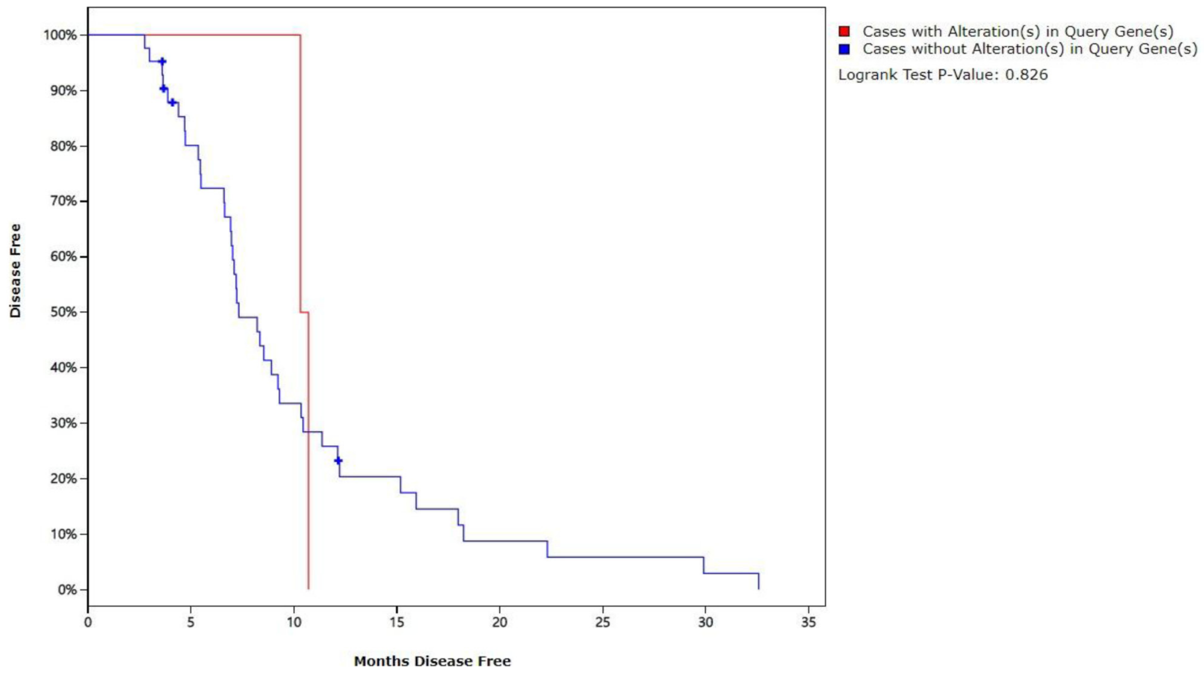

B

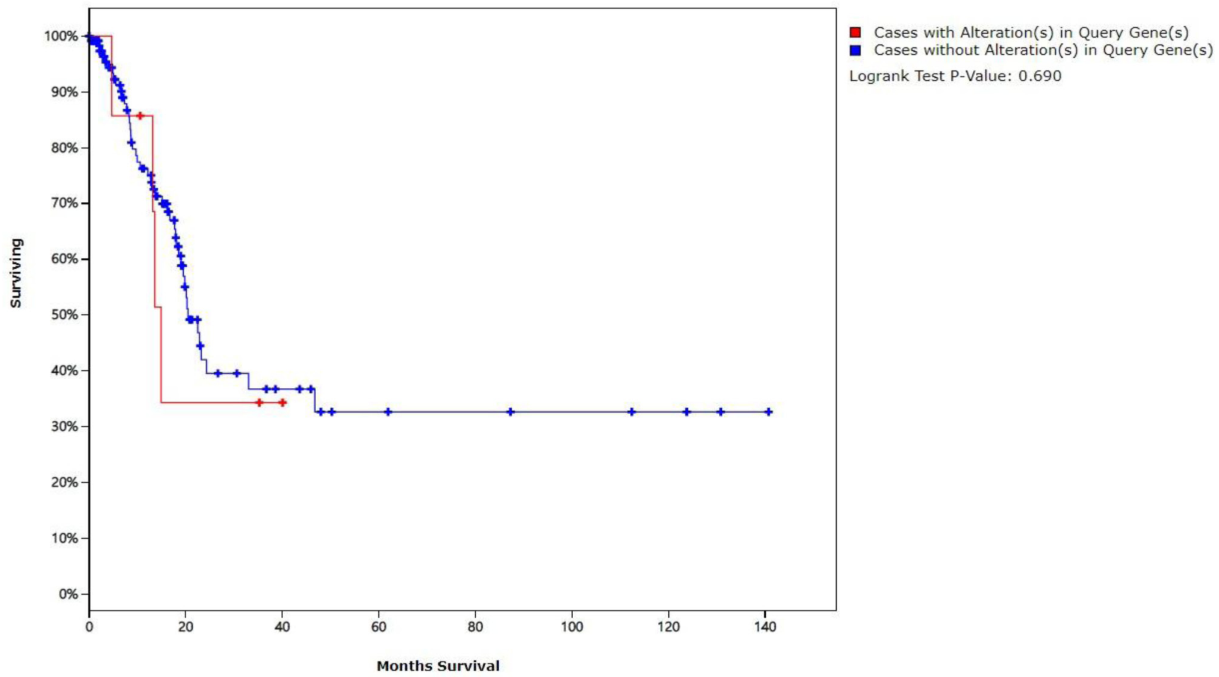

Supplementary Figure 3: Kaplan–Meier survival curves for disease-free survival (**A**) and overall survival (**B**) according to HSPB1 gene expression in an independent TCGA database including muscle-invasive bladder cancers [32].

Supplementary Table 1: Baseline characteristics of a validation cohort comprising 132 patients with primary non-muscle invasive bladder

Variables	No. (%)
Gender	
Male	107 (81.1)
Female	25 (18.9)
Tumor size	
<3 cm	94 (71.2)
≥3 cm	38 (28.8)
Multifocality	
Single	100 (75.8)
Multiple	32 (24.2)
Concomitant carcinoma-in-situ	
No	110 (83.3)
Yes	22 (16.7)
Morphology	
Papillary	117 (88.6)
Sessile	15 (11.4)
Lymphovascular invasion	
No	108 (81.8)
Yes	24 (18.2)
Intravesical therapy	
No	71 (53.8)
Yes	61 (46.2)
Tumor stage	
Ta	57 (43.2)
T1	75 (56.8)
Grade	
Low	77 (58.3)
High	55 (41.7)
Recurrence	
No	86 (65.2)
Yes	46 (34.8)
Progression	
No	113 (85.6)
Yes	19 (14.4)
